# Supplementary material for: Coupling sensor to enzyme in the voltage sensing phosphatase
Source: Nat Commun. 2024 Jul 30;15:6409. doi: 10.1038/s41467-024-50319-8 (PMC11289409; doi:10.1038/s41467-024-50319-8)
Supplement: Supplementary file 1 — Supplementary Information [file 41467_2024_50319_MOESM1_ESM.pdf]

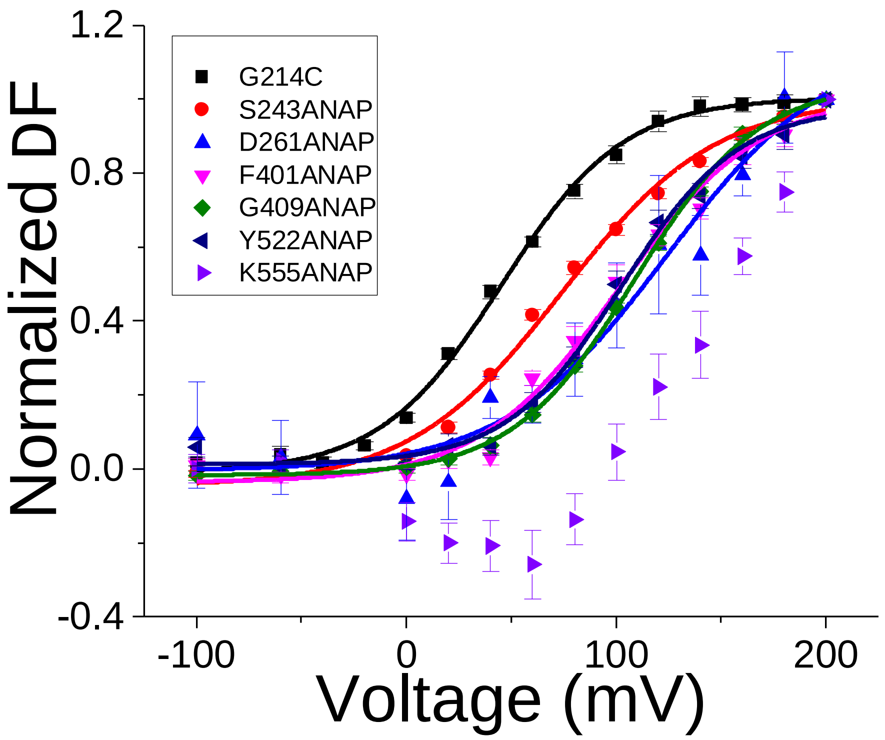

Figure S1. Voltage-dependence of S4 and PD rearrangements. Comparison of voltage dependence of ANAP fluorescence when incorporated at different sites as indicated. F-V curves of ANAP fluorescence were obtained using Protocol 1 (See Methods). Fluorescence measured at the end of each step voltage was normalized to that obtained at 200 mV. Fluorescence traces were fit with Boltzman equations and $V_{1/2}$ for TMRM or ANAP fluorescence at each site are as follows: G214C-TMRM 45 ± 2 mV (n = 12), S243ANAP 74 ± 4 mV (n = 14), D261ANAP 124 ± 17 mV (n = 5), F401ANAP 101 ± 5 mV (n = 10), G409ANAP 109 ± 1 mV (n = 16), Y522ANAP 102 ± 4 mV (n = 12).

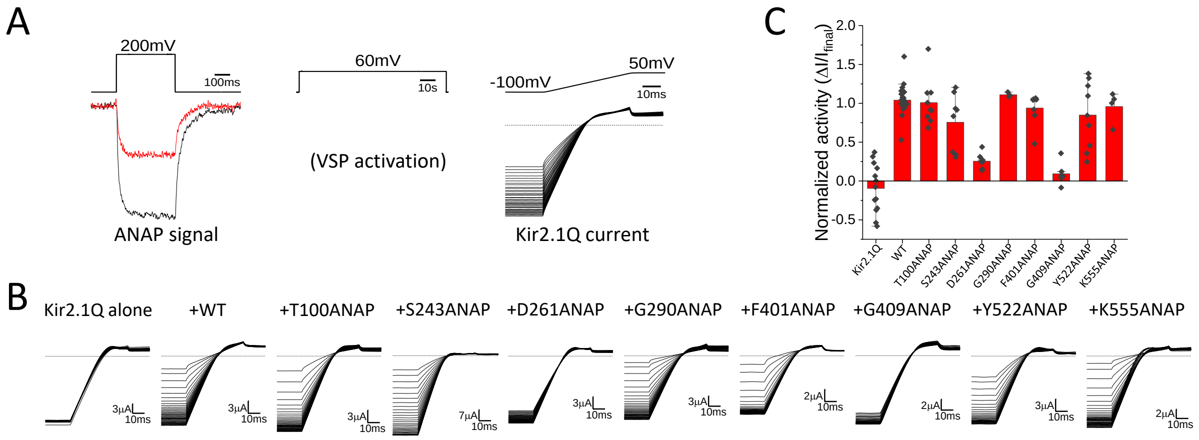

Figure S2. VSP remains functional with ANAP incorporated in linker, near R loop, gating loop, catalytic site and C2 domain. (**A**) Voltage protocols and recording traces for ANAP incorporation detection, VSP activation (PI(4,5)P_2 depletion) and Kir2.1Q current monitor (See Methods). (**B**) Representative Kir2.1Q current traces recorded when expressed alone or co-expressed with different ANAP-incorporated Ci-VSPs. (**C**) Comparison of activities between WT and different ANAP-incorporated Ci-VSPs (n = 3 to 21). Calculated activity from each condition was normalized to that of WT.

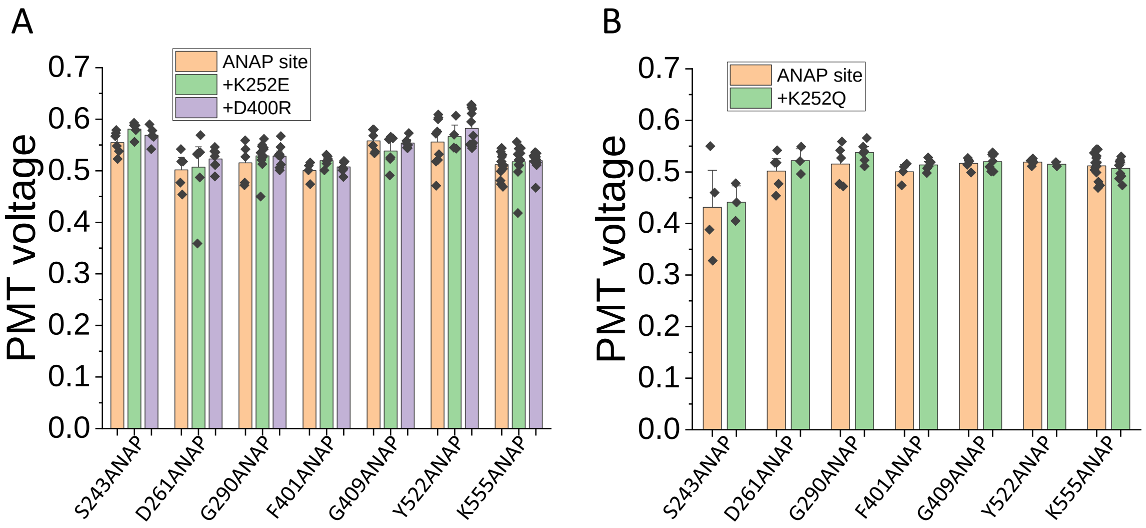

Figure S3. Expression level of ANAP-incorporated Ci-VSPs remains similar with mutation K252E, K252Q and D400R. (**A, B**) Comparison of expression level of Ci-VSPs when ANAP-incorporated at each site to that with mutation K252E (**A**), D400R (**A**) and K252Q (**B**) based on the TMRM labeling on G214C (n = 4 to 15). The Y axis refers to the voltages of the photomultiplier tube required to bring the baseline fluorescence to the same level at the holding voltage. Equal PMT voltages mean equal fluorescence intensities and indicate similar surface expression levels[1].

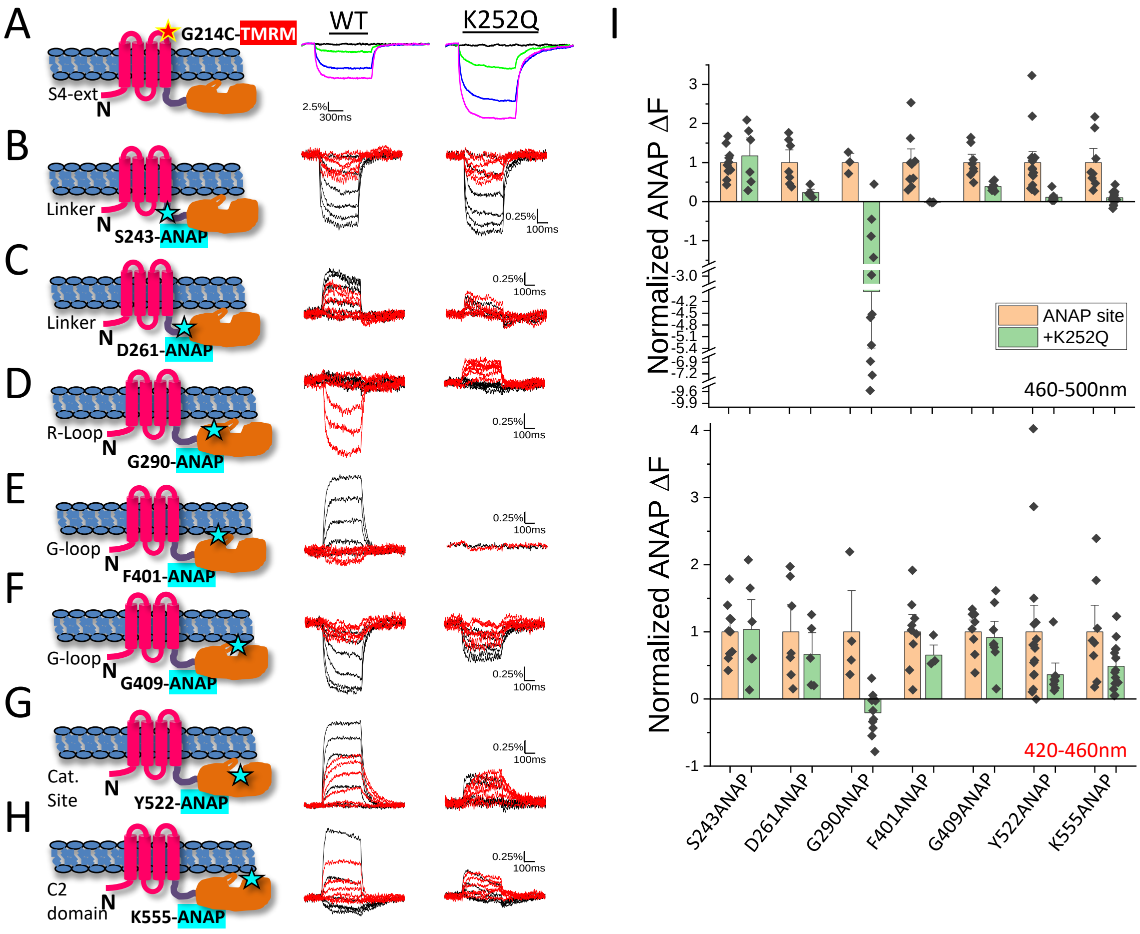

Figure S4. Mutation of K252Q also strongly affects the voltage-driven conformational changes in the linker, R loop, gating loop and catalytic site. (**A**) Representative fluorescence traces from TMRM-labeled G214C of WT Ci-VSP and with K252Q at 200 mV (magenta), 80 mV (blue), 20 mV (green) and -100 mV (black). (**B-H**) Superposition of representative fluorescence traces from ANAP incorporated at proximal linker 243 (**B**), distal linker 261 (**C**), near R-loop 290 (**D**), gating loop 401 (**E**) and 409 (**F**), catalytic site 522 (**G**) and C2 domain 555 (**H**). Fluorescence was collected at 460-500 nm (black) and 420-460 nm (red). Voltage steps are 0 mV, 40 mV, 80 mV, 120 mV, 160 mV and 200 mV. (**I**) Comparison of fluorescence amplitude of ANAP at different sites to that with K252Q from 460-500 nm (top) and 420-460 nm (bottom) (n = 4 to 15). The cartoon in each panel indicates the individual labeling site. Recordings on individual sites with and without mutations were done on the same batch of oocytes and on the same day, and the same batch of injected oocytes were labeled with TMRM under the same conditions. The scales of X and Y axis are the same in each ANAP labeling site and with K252Q.

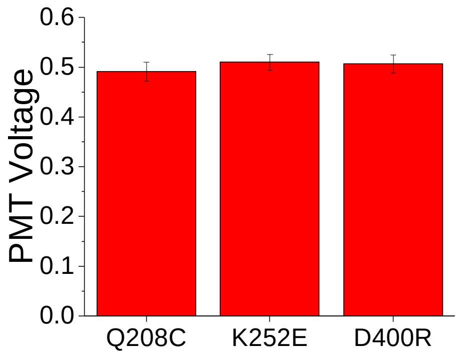

Figure S5. Mutation of K252E and D400R expressed similarly to WT in the enzyme activity assay. Comparison of expression level of WT to that with K252E or D400R mutation in the enzyme activity assay. The same batch of injected oocytes used for activity assay was labeled with TMRM on Q208C for expression assessment (n = 11 to 12).

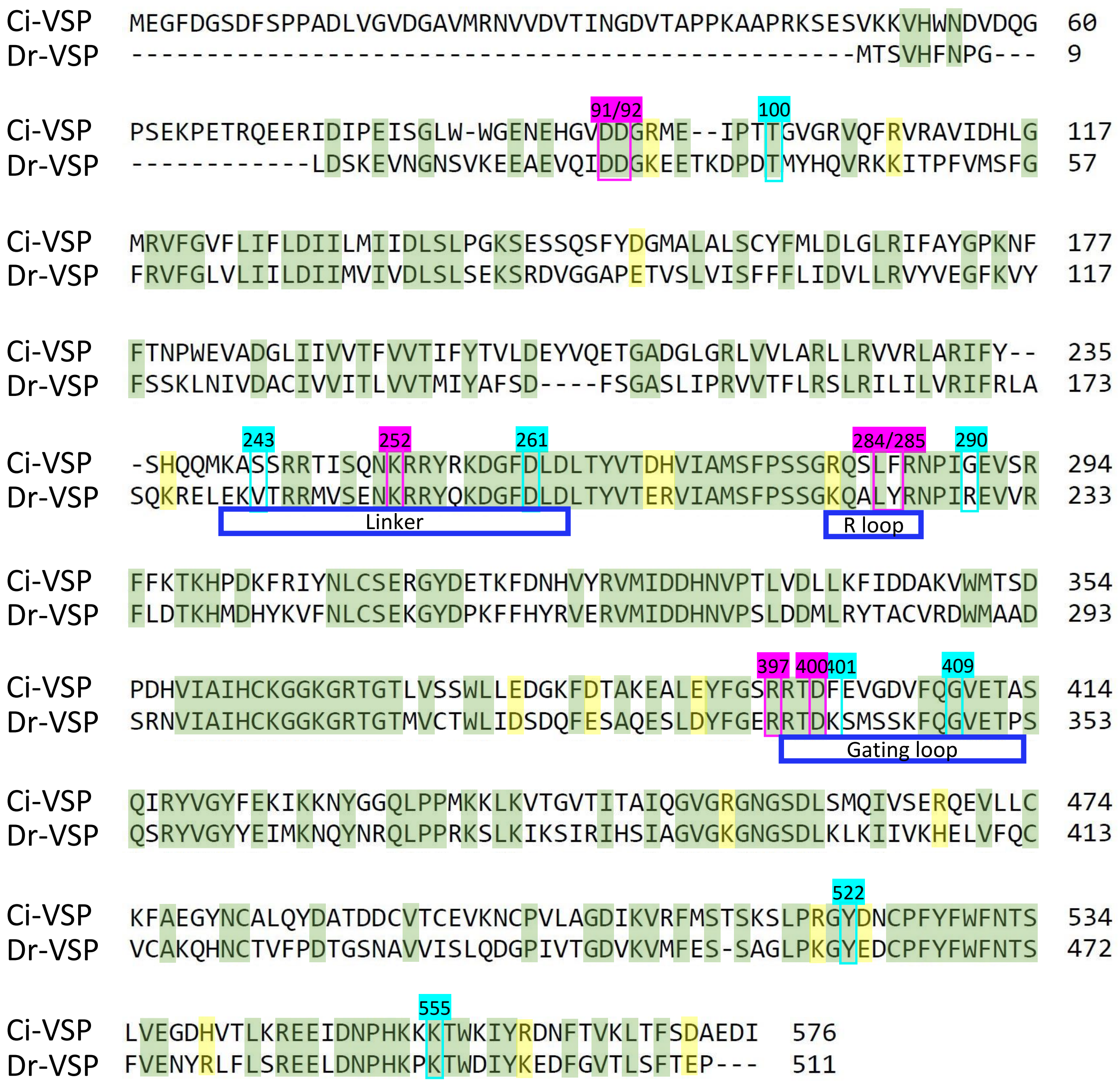

Figure S6. Protein sequence alignment between Ci-VSP and Dr-VSP. Sites used for ANAP labeling are in cyan and residues tested in the experiments are in magenta. Regions are labeled at the bottom of the corresponding sequence. Residues are numbered based on Ci-VSP sequences. Identical residues are highlighted green and conserved charged residues are highlighted yellow.

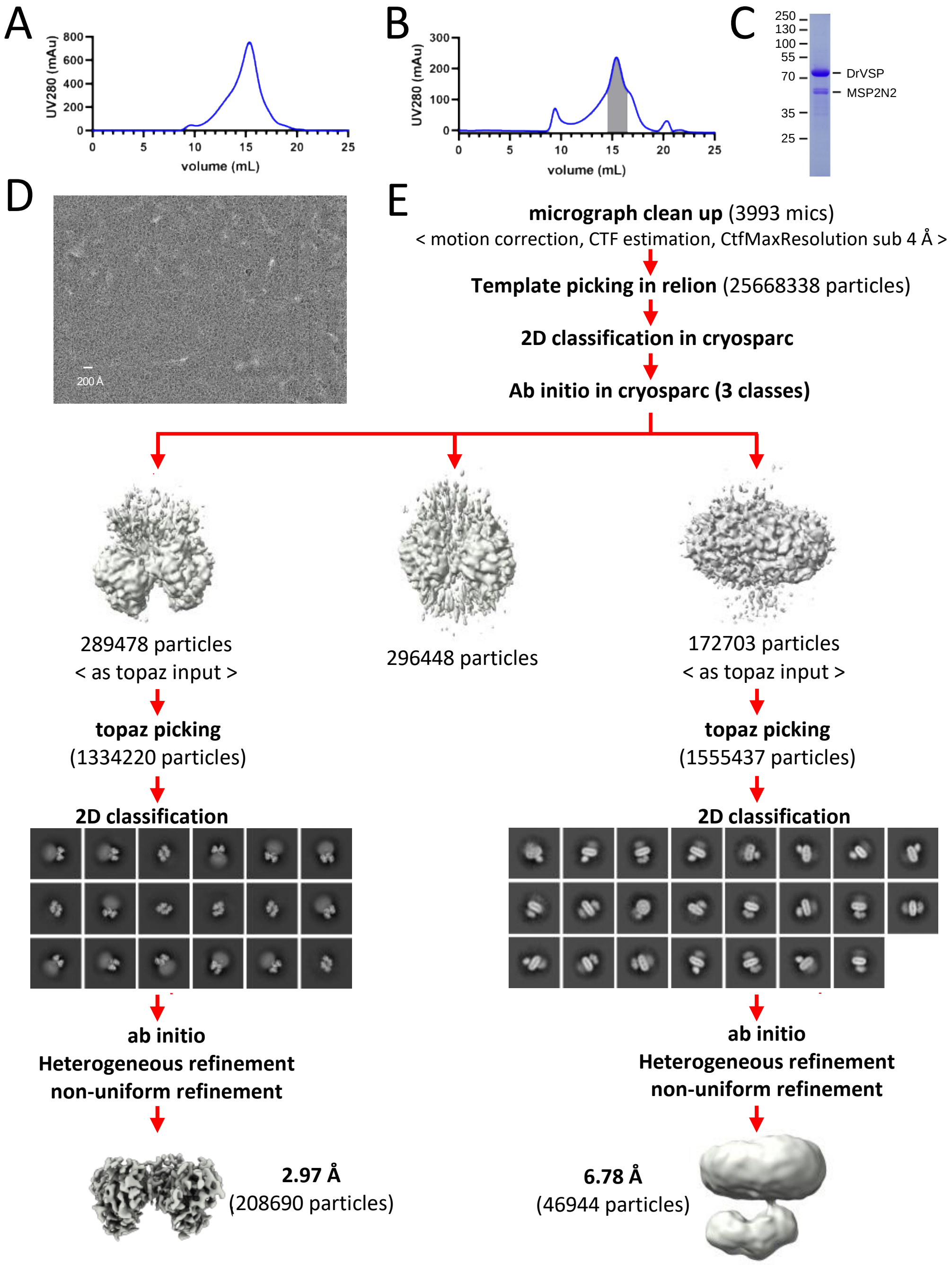

Figure S7. Biochemistry and cryo-EM data processing of Dr-VSP. (**A**, **B**) Size-exclusion chromatogram showing the purification (**A**) and reconstitution of Dr-VSP in lipid nanodiscs (**B**). Shaded region corresponds to the pooled fractions collected for cryo-EM sample preparation. Samples were run on a Superose 6 column. (**C**) Coomassie stained SDS-PAGE gel of the pooled fractions collected for cryo-EM sample preparation. (**D**) Representative cryo-EM micrograph of Dr-VSP in lipid nanodiscs (scale bar = 200 Å). (**E**) See methods for cryo-EM data processing details.

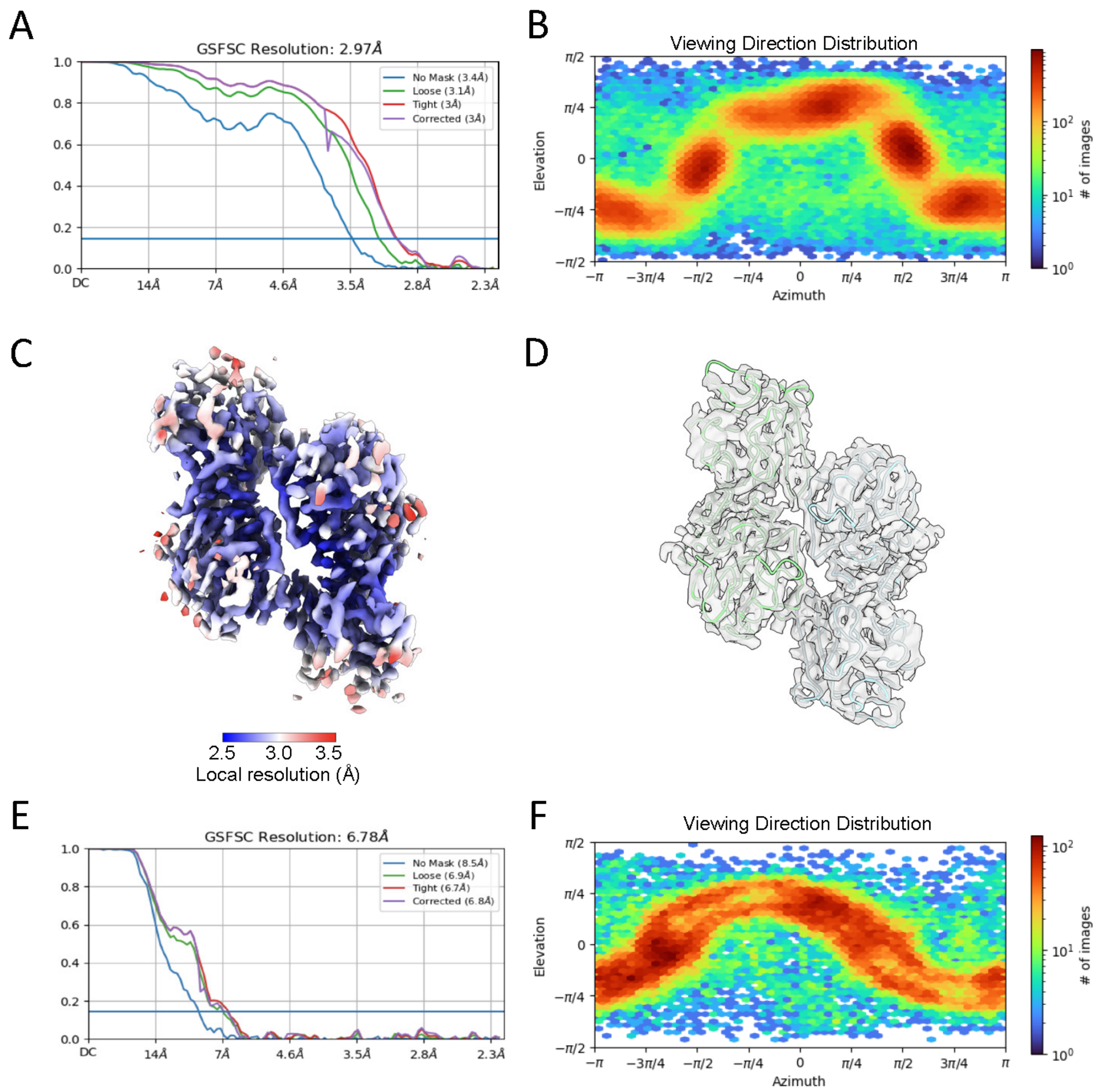

Figure S8. Cryo-EM validation of the dimeric PD domain and monomeric Dr-VSP in nanodiscs. (**A)** Fourier Shell Correlation (FSC) curve and **(B)** angular distribution of particles from the final dimeric PD domain reconstruction. (**C**) Cryosparc-estimated local resolution colored as indicated and **(D)** final PD domain model fit in map. (**E**) FSC curve and **(F)** angular distribution of particles from the final monomeric Dr-VSP reconstruction.

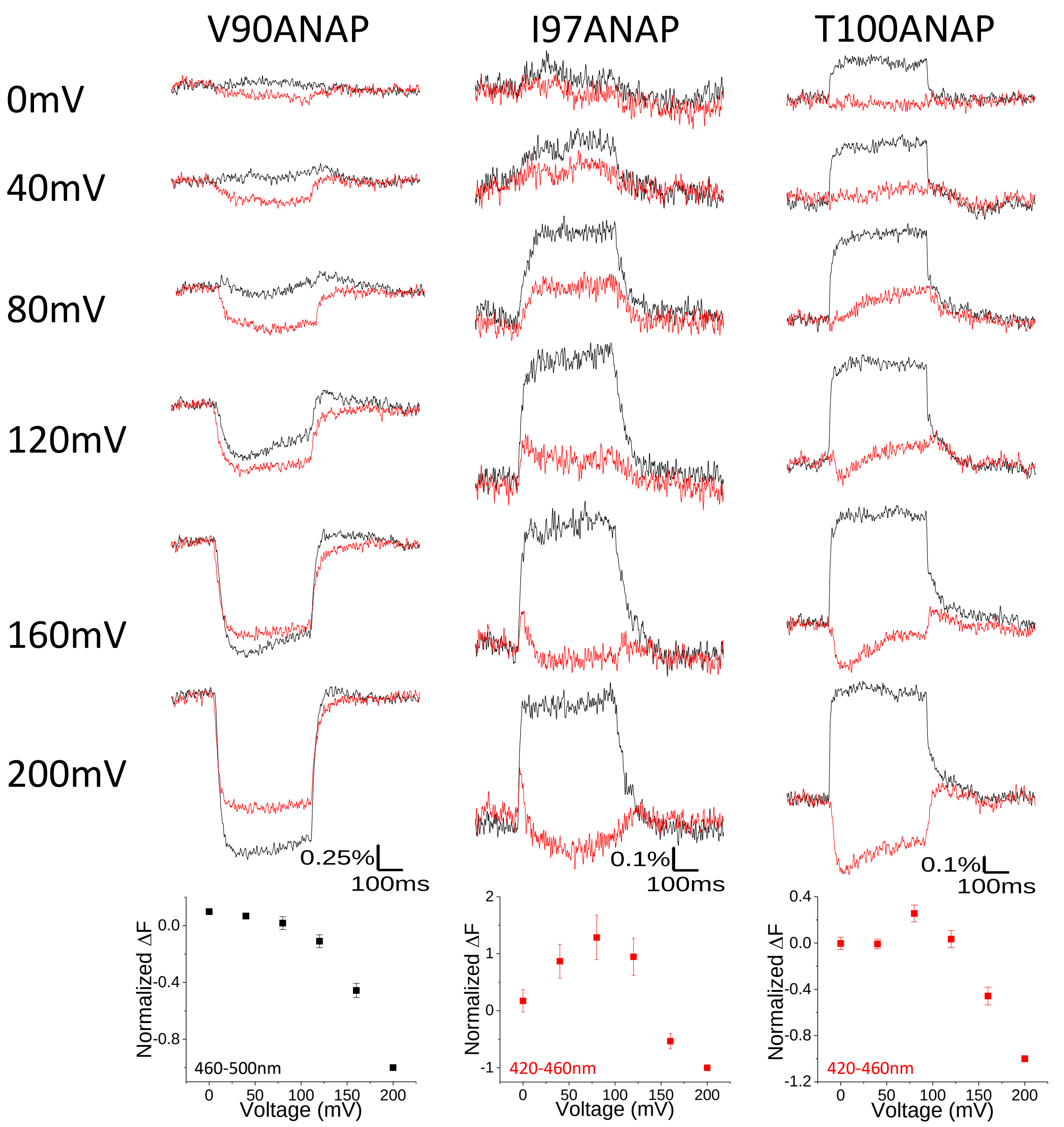

Figure S9. Some N-terminus residues show biphasic voltage-dependent conformational changes. Representative fluorescence traces of ANAP incorporated at N-terminus site 90, 97 and 100 at voltages as indicated. Fluorescence was simultaneously recorded from 460-500 nm (black) and 420-460 nm (red). F-V curves of ANAP fluorescence at different sites, collected from the indicated wavelength range, are displayed at the bottom of corresponding traces. The F-V curves Fluorescence measured at the end of the test pulses was normalized to the one at 200 mV. (n = 4 to 12)

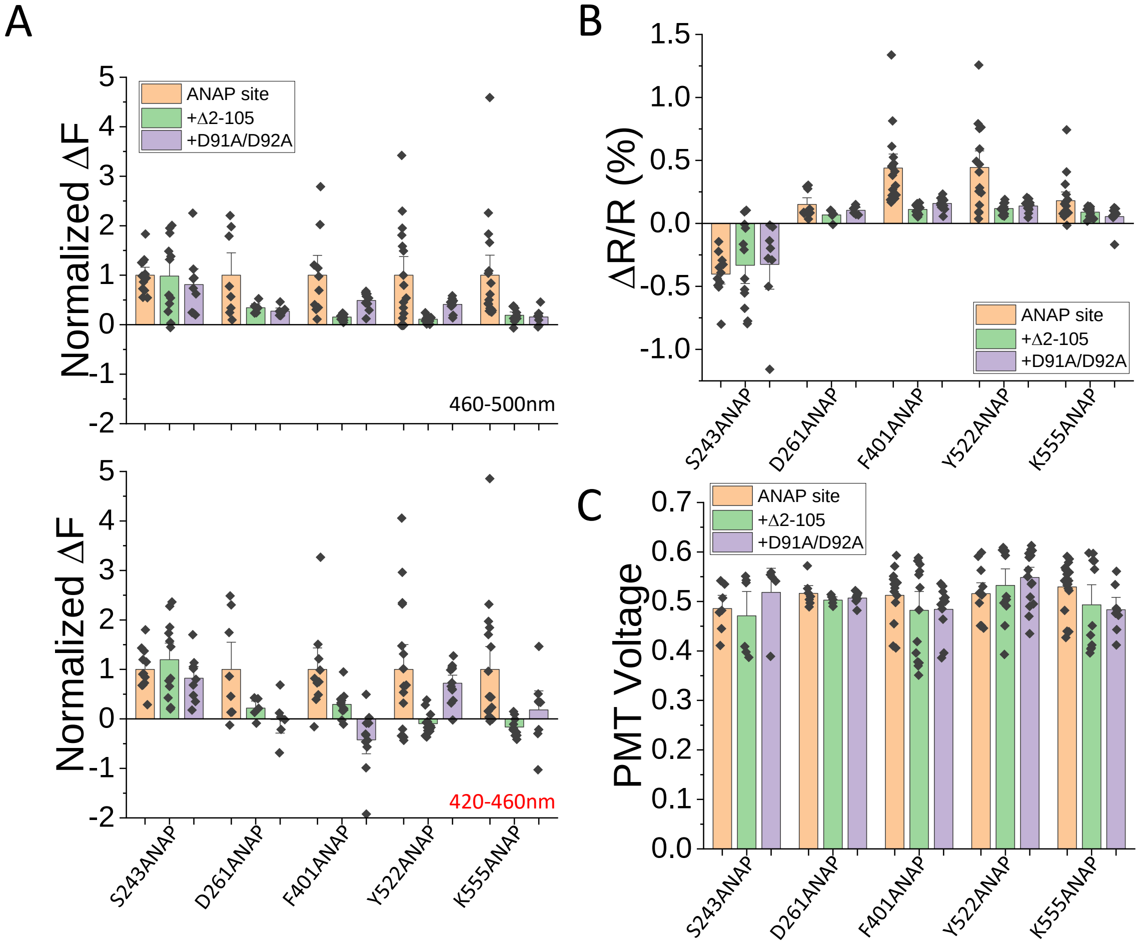

Figure S10. Comparison of ANAP ΔF amplitude, spectral shift and expression level of WT and that with N-terminus deletion Δ2-105 and D91A/D92A mutation. (**A**) Comparison of ANAP ΔF amplitude at different sites to that with N-terminus deletion Δ2-105 and with D91A/D92A mutation from 460-500 nm (top) and 420-460 nm (bottom) (n = 8 to 27). (**B**) Comparison of spectral shift upon activation in WT to that with Δ2-105 and with D91A/D92A (n = 8 to 27). ΔR/R measures the change of fluorescence ratio between the 460-500 nm and the 420-460 nm emission channels when VSP is activated at 200 mV (See Methods). (**C**) Comparison of expression level of Ci-VSPs when ANAP-incorporated at each to that with Δ2-105 and with D91A/D92A based on the TMRM labeling on G214C (n = 5 to 18). Recordings on individual sites with and without mutations were done on the same batch of oocytes and on the same day, and the same batch of injected oocytes were labeled with TMRM under the same conditions.

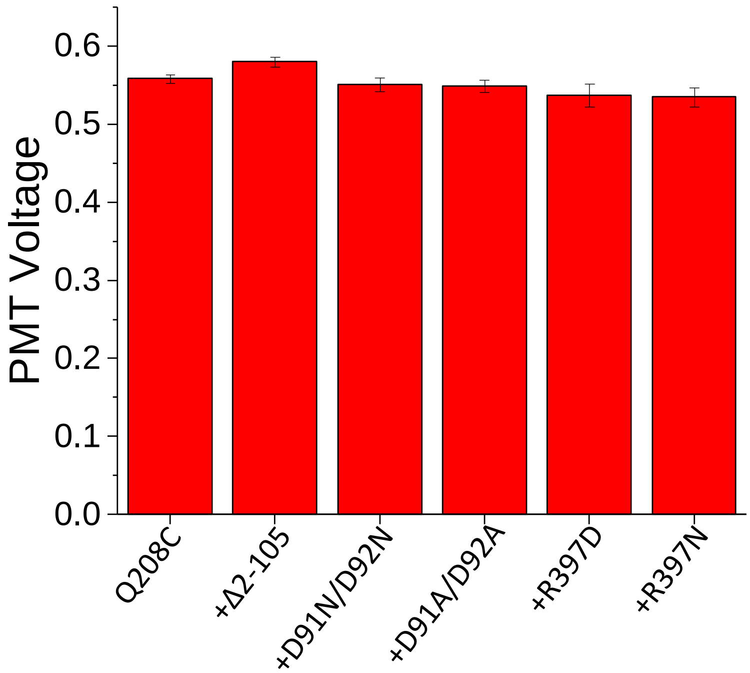

Figure S11. Comparison of expression level of WT and mutants tested in the enzyme activity assay. The same batch of injected oocytes used in the enzyme activity assay was labeled with TMRM on Q208C for expression assessment (n = 11 to 20).

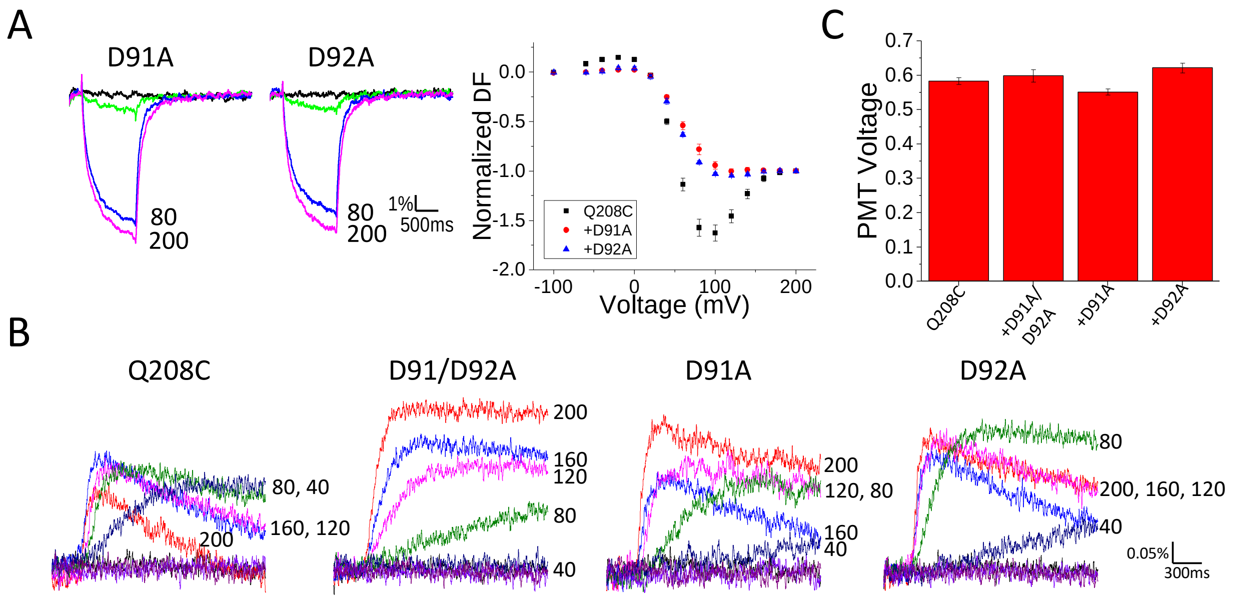

Figure S12. Both D91A and D92A contribute to the effect of D91A/D92A mutation. (**A**) Representative fluorescence traces and F-V of TMRM-labeled Q208C of D91A and D92A (n = 12 to 14). Traces recorded at 200 mV (magenta), 80 mV (blue), 20 mV (green) and -100 mV are shown. (**B**) Representative FRET traces of F-TAPP co-expressed with WT and different mutants using 2-s test protocol (See Methods). Some test voltages are shown next to the corresponding traces. (**C**) Comparison of expression level of WT to that with D91A and D92A in the enzyme activity assay (n = 15 to 26). The same batch of injected oocytes used in the enzyme activity assay was labeled with TMRM on Q208C for expression assessment.

	Dr-VSP PD domain
**Data collection and processing**	
magnification	36,000
voltage (kV)	200
electron exposure (e^{-} Å^2)	50
defocus range (μm)	-0.6 to -1.8
pixel size (Å)	0.5575
symmetry imposed	
initial particle images (no.)	25668338
final particle images (no.)	208690
map resolution (Å)	2.97
FSC threshold	0.143
**Refinement**	
model resolution (Å)	2.94
FSC threshold	0.143
map sharpening B factor (Å^2)	
model composition	
non-hydrogen atoms	4760
protein residues	586
B factors (Å^2)	
protein	49.17
r.m.s deviations	
bond lengths (Å)	0.014
bond angles (Å)	1.348
validation	
MolProbity score	2.32
clash score	17.91
poor rotamers (%)	1.13
Ramachandran plot	
favored (%)	90.83
allowed (%)	8.65
disallowed (%)	0.52

Supplemental Table-1. Cryo-EM data collection, refinement and validation statistics.

Supplemental References

1. Kohout, S.C. et al. Electrochemical coupling in the voltage-dependent phosphatase Ci-VSP. *Nat Chem Biol* **6**, 369-75 (2010).
2. Liu, L. et al. A glutamate switch controls voltage-sensitive phosphatase function. *Nat Struct Mol Biol* **19**, 633-41 (2012).
