# Supplementary material for: Coupling sensor to enzyme in the voltage sensing phosphatase
Source: Nat Commun. 2024 Jul 30;15:6409. doi: 10.1038/s41467-024-50319-8 (PMC11289409; doi:10.1038/s41467-024-50319-8)
Supplement: Supplementary file 3 — Description of Additional Supplementary Files [file 41467_2024_50319_MOESM3_ESM.pdf]

Description of Additional Supplementary Files

File Name: Supplementary Movie 1

Description: Movie made from superposing different crystal structures of the isolated cytosolic domain. The crystal structures of chain a and b of 3V0F, chain a of 3V0D and chain a of 3V0H2 were used to construct the movie. The gating loop is labeled purple and the R loop orange. The side chain of E411 is shown.

File Name: Supplementary Movie 2

Description: Movie showing the AlphaFold-predicted model of CiVSP. The same regions and side chains are labeled as shown in Figure 3A inset.

File Name: Supplementary Movie 3

Description: Movie comparing the crystal structure of Ci-VSP PD (form II, PDB: 3V0F2) (yellow) and the high-resolution cryo-EM structure of Dr-VSP PD (gray).

File Name: Supplementary Movie 4

Description: Movie showing the cryoEM density map of the dimeric DrVSP.
